# Autophagy-related gene-based prognostic model for breast cancer

**DOI:** 10.1007/s12672-026-04771-1

**Published:** 2026-03-07

**Authors:** Pei Huang, Zhenbang Lie, Weichao Yang, Xin Li

**Affiliations:** 1 Department of Oncology, Dongguan Kanghua Hospital, No. 1000, Dongguan Avenue, Nancheng Street, Dongguan, Guangdong China; 2 Department of Cardiology, Dongguan People’s Hospital, Dongguan, Guangdong China

**Keywords:** Autophagy-related genes (ARGs), Breast cancer (BRCA), Neoadjuvant chemotherapy (NAC), Prognostic, Drug sensitivity, Immune microenvironment

## Abstract

**Purpose:**

Breast cancer (BRCA), a major global health issue, has recently become the first major cancer, surpassing lung cancer, and the primary contributor to female cancer deaths. Neoadjuvant chemotherapy (NAC) serves as the standard treatment for BRCA and is associated with favorable disease-free survival (DFS) and overall survival (OS). Autophagy-related genes (ARGs) not only facilitate tumor initiation and progression but also exhibit a close correlation with chemoresistance. This study intends to explore the molecular signature for predicting the BRCA immune response and prognosis based on ARGs.

**Methods:**

We collected RNA sequencing data of BRCA from The Cancer Genome Atlas (TCGA) and Gene Expression Omnibus (GEO), and screened prognostic genes by WCGNA, univariate Cox analysis, and LASSO regression. Then, based on 11 chemotherapy-sensitive ARGs (C-ARGs), a prognostic model was established using a multivariate Cox analysis. Subsequently, to assess the model’s performance (TCGA), we plotted receiver operating characteristic (ROC) curves and conducted external validation (GEO).

**Results:**

We found that for 1-/3-/5-year overall survival, the areas under the ROC curve (AUCs) were 0.654, 0.673, and 0.698 in the TCGA training cohort, and 0.641, 0.719, and 0.749 (GSE42568) and 0.635, 0.742, and 0.702 (GSE20685) in the GEO validation cohort. The results demonstrated the satisfactory performance of the C-ARG-based model in predicting the efficacy and BRCA prognosis. In addition, in the low-/high-risk groups, the proportion of immune subtypes and the IC50 value significantly differed, which can guide the systemic therapy.

**Conclusion:**

The C-ARG-based prognostic model exhibits excellent performance in predicting the BRCA prognosis, which lays a solid foundation for the development of clinical treatment protocols for BRCA.

**Supplementary Information:**

The online version contains supplementary material available at 10.1007/s12672-026-04771-1.

## Introduction

 Breast cancer (BRCA) is an important health issue worldwide, and it has recently become the most frequent type of cancer, surpassing lung cancer, and the primary cause of female cancer death, as revealed by the Global Cancer Statistics 2020 [1]. As various treatment (e.g., chemotherapy, surgery, radiotherapy, endocrine therapy, targeted therapy) constantly develop, the survival rate of BRCA patients has been raised. Neoadjuvant chemotherapy (NAC) has become the standard treatment for early high-risk and locally advanced BRCA, which can lower the stage and reduce the volume of the tumor to conserve the breast. Achievement of pathological complete response (pCR) after NAC is generally associated with a considerable improvement in both overall survival (OS) and disease-free survival (DFS) among BRCA patients [2]. Despite substantial progress in the BRCA treatment, some challenges remain unresolved, such as chemoresistance, lack of sufficient molecular targets, and undetected distant metastases. Besides, the chemosensitivity and prognosis of some patients are still unsatisfactory due to individual heterogeneity [3]. To improve clinical management, therefore, it is necessary to develop accurate strategies for predicting chemosensitivity, long-term prognosis, and tumor and immune microenvironment. Comprehensive models integrating multiple key characteristics are seemingly more reliable for prognostic prediction than single indicators (e.g., clinicopathologic parameters or individual gene expressions).

First discovered by Ashford and Porter in 1962, autophagy, also known as “self-eating”, is a highly conserved and pervasive physiological process during evolution [4]. In response to internal or external metabolic stimuli such as starvation and hypoxia, autophagy is initiated to maintain homeostasis in the body by delivering aggregated proteins, excess or damaged organelles, and pathogens to lysosomes for hydrolysis and degradation into small molecules [5]. Recent research suggests that autophagy is linked to both cancer development and treatment, and it exerts complex effects on cancers, i.e., it prevents bioenergetic failure caused by metabolic stress and controls the quality and quantity of proteins and organelles, and also contributes to tumorigenesis and maintenance of the malignant state [6]. Autophagy can play dual roles (anti- or pro-tumor) at different periods of cancer development due to a single or multiple effects of tumor microenvironment (TME) stress, pathogenic conditions, and immune system [7, 8], whose exact mechanism remains to be investigated. As revealed by numerous basic experiments and clinical trials, the high expression of autophagy-related genes (ARGs) is closely linked to tumor development and adverse prognosis [9, 10]. Furthermore, autophagy influences the tolerance of tumor cells to chemotherapeutic agents via multiple mechanisms, including intracellular signaling pathways and regulation of the tumor microenvironment, making it an important molecular mechanism for chemotherapy efficacy and chemoresistance [11, 12]. Currently, most ARGs prognostic models for breast cancer focus on prognostic and predictive models for breast cancer recurrence and metastasis [13–15], however, rare studies are available on the role of ARGs in chemosensitivity and prognosis of BRCA.

Therefore, this study established a chemotherapy-sensitive ARG (C-ARG)-based prognostic model using the sequencing data from NAC for BRCA, and underwent external validation in the Gene Expression Omnibus (GEO) dataset. Then we further investigated the association of the model signature with the immune microenvironment. We found that this C-ARG-based prognostic model well predicted the efficacy and BRCA prognosis, and also provided individualized immunotherapy with clinical guidance (Table 1)

## Methods

### Data

Relevant data were acquired from The Cancer Genome Atlas (TCGA) (http://xena.ucsc.edu/), including transcriptome RNA-seq and clinical data. After 23 samples were excluded due to no prognostic information and 29 due to no data on the gene expression, 1203 BRCA samples were included. We downloaded the datasets GSE25065, GSE20685, and GSE42568 from the GEO (https://www.ncbi.nlm.nih.gov/geo/). GSE25065 was generated by the GPL96 (Affymetrix Human Genome U133A Array) and contained 182 BRCA samples. GSE20685 and GSE42568 were generated by the GPL570 (Affymetrix Human Genome U133 Plus 2.0 Array). After data without prognostic information were excluded, GSE20685 contained 327 BRCA samples, while GSE42568 contained 104 BRCA samples. GSE25065, GSE20685, and GSE42568 all underwent normalization. C-ARGs for BRCA were identified by WGCNA in GSE25065, and a prognostic model was established in TCGA-BRCA and externally validated in GSE20685 and GSE42568. As of January 22, 2025, we retrieved 1620 ARGs (http://www.tanpaku.org/autophagy/).

### WGCNA

We created a co-expression network using WGCNA in GSE25065 to explore the modules highly linked to pCR in NAC for BRCA. First, we filtered 25% of the data on gene expression to remove some low-expression or noisy genes. A clustering tree was constructed to detect outliers, and the soft threshold power was determined by network topology analysis. Subsequently, the adjacency relation was assessed and transformed into a topological overlap matrix. We calculated the diversity factor and generated a hierarchical clustering tree for genes. Modules with similar expression patterns were identified and combined by a dynamic tree-cutting approach, and genes in the key modules most relevant to pCR were identified and intersected with ARGs.

### Protein-protein interaction (PPI) and enrichment analyses

PPI analysis can better explain the relation of different proteins at the genome level and offer a new perspective for protein functional interpretation. A PPI network was constructed for the intersection genes (IGs) by the STRING (v12.0, https://string-db.org/) covering 12,535 species and 59.3 million proteins, and then visualized by Cytoscape.

Meanwhile, the IGs underwent Gene Ontology (GO) and Kyoto Encyclopedia of Genes and Genomes (KEGG) analyses by the R clusterProfiler package. P_corrected_<0.05 suggested statistical significance.

### Construction and validation of a C-ARG-based prognostic model

C-ARGs were first screened by univariate Cox proportional hazards regression in TCGA, with the significance level set at *P* < .05, to preliminarily identify gene sets potentially with prognostic value. To address potential overfitting in univariate analysis and enhance the stability and robustness of the prognostic signature, the C-ARGs preliminarily screened were further subjected to dimensionality reduction and optimization by LASSO Cox proportional hazards regression analysis to identify hub genes. During the LASSO regression, model performance was assessed by 10-fold cross-validation to select the gene subset with optimal predictive performance. Finally, a multivariate Cox proportional hazards regression model was created based on the hub genes identified by LASSO regression, and the risk score was calculated as follows to quantify the prognostic risk: Riskscore = h_0_(t) × exp(∑Coef_i_ × x_i_), where xi represents the normalized expression of target gene i, and Coef_i_ represents the regression coefficient.

Based on the median of risk score, the TCGA-BRCA samples were assigned to high-/low-risk groups, and underwent Kaplan-Meier and log-rank tests for the OS. To assess the predictive accuracy, we drew time-dependent receiver operating characteristic (ROC) curves (R timeROC package), and the AUCs for 1-/3-/5-year OS were compared, followed by external validation in GSE42568 and GSE20685.

### Construction of a nomogram

Nomograms have been widely applied to prognostic prediction, which is constructed by integrating independent prognostic factors, such as risk scores and clinical characteristics. We calculated nomogram scores for the 1-/3-/5-year OS in BRCA and assessed the calibration capability by decision curve analysis (DCA) and calibration curves.

### Immune infiltration and immune function analyses

To further analyze the association of immune cell signature with the risk, we assessed the immune infiltration in TCGA-BRCA samples using CIBERSORT, analyzed the content of immune infiltrating cells by Wilcoxon signed-rank tests, and explored the correlation of immune infiltrating cells with genes. The results were all visualized by the R ggplot2 package. Meanwhile, downloaded from the TCIA (https://tcia.at/), the immunophenotype score (IPS) allowed specific prediction of the patient’s response to two immune checkpoint (IC) inhibitors (anti-*CTLA-4/PD-1*). Single-sample gene set enrichment analysis (GSEA) was also performed (clusterProfiler).

The TME scores (Immune, Stromal, and ESTIMATE) were calculated and compared by the ESTIMATE package. We also compared the expression of human leukocyte antigen (HLA) and ICs, and performed mutation analyses in the two groups.

### Drug sensitivity assessment

The IC50 value of common chemotherapeutic and targeted drugs was calculated by the R pRRophetic package, based on which the drug sensitivity was compared by Wilcoxon signed-rank tests.

### Immunohistochemistry (IHC) staining

We compared the protein expression of C-ARGs involved in the prognostic model between normal breast tissues and BRCA tissues using IHC images downloaded from the HPA (http://www.proteinatlas.org/) and also at UALCAN (https://ualcan.path.uab.edu/index.html).

### Statistical analysis

R (v4.4.2) was utilized for data analysis. We screened prognostic factors by univariate/multivariate Cox analyses, and the model was assessed for the OS predictive value by the Kaplan-Meier test and for the predictive accuracy by ROC curves. *P* < .05 was deemed statistically significant.

## Results

### WCGNA

**Fig. 1:**
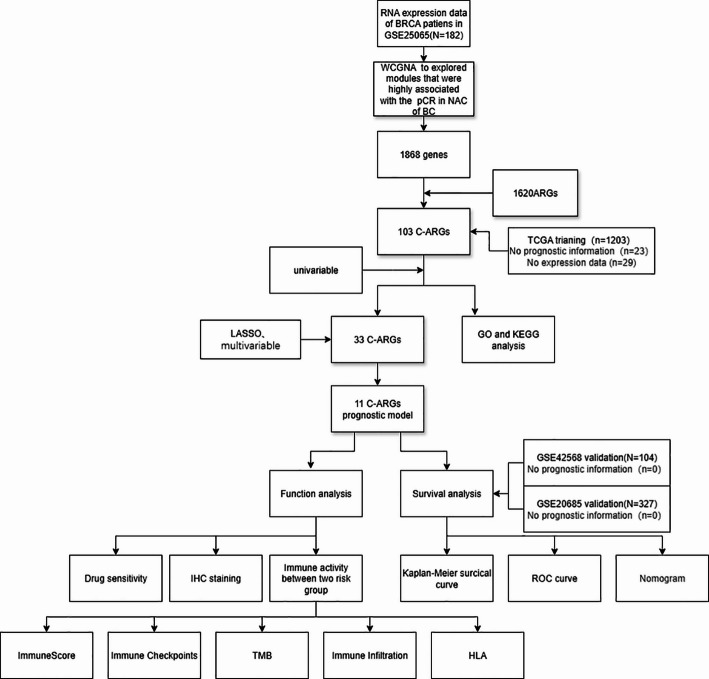
Flow diagram of the study

**Fig. 2:**
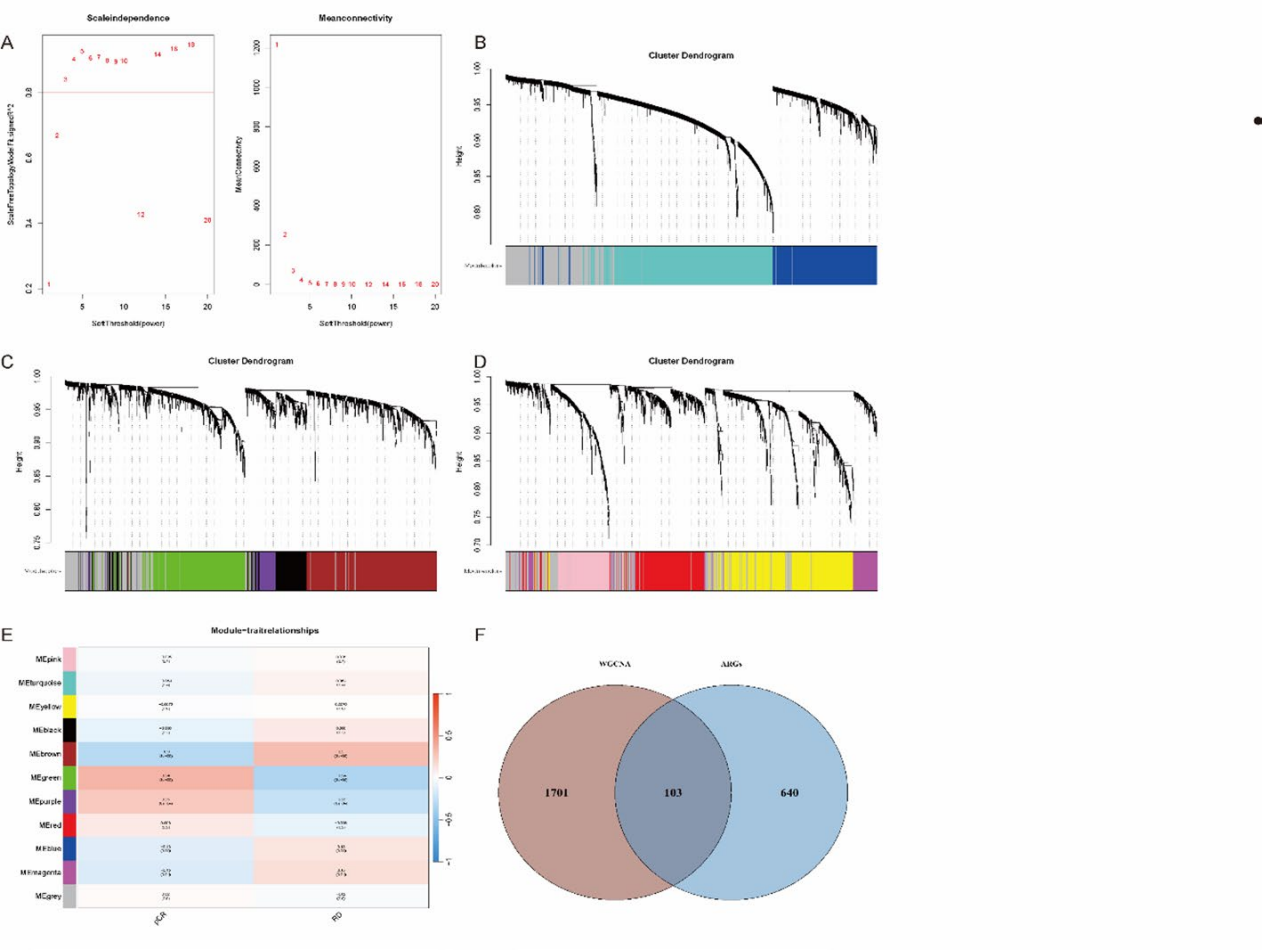
WCGNA used to explore modules highly associated with the pCR in NAC of GSE25065: **A** Soft thresholding; **B**–**D** Cluster Tree; **E** Module-trait relationships; **F** Venn diagram of prognostic

The study flowchart is displayed in Fig. 1. First, we constructed a clustering tree, and no outliers were found. Then we determined a soft threshold power of 3 by a network topology analysis (Fig. 2A), based on which the genes were categorized into 11 modules (Fig. 2B-D). The module-pCR heatmap revealed that three modules, MEgreen, MEpurple, and MEbrow, had the closest association with pCR, with correlation coefficients of 0.34, 0.25, and − 0.3 (all *P* < .001), respectively (Fig. 2E). Finally, we screened 1868 eligible hub genes and intersected them with ARGs, from which 103 IGs were acquired (Fig. 2F).

### PPI and functional enrichment analyses

**Fig. 3:**
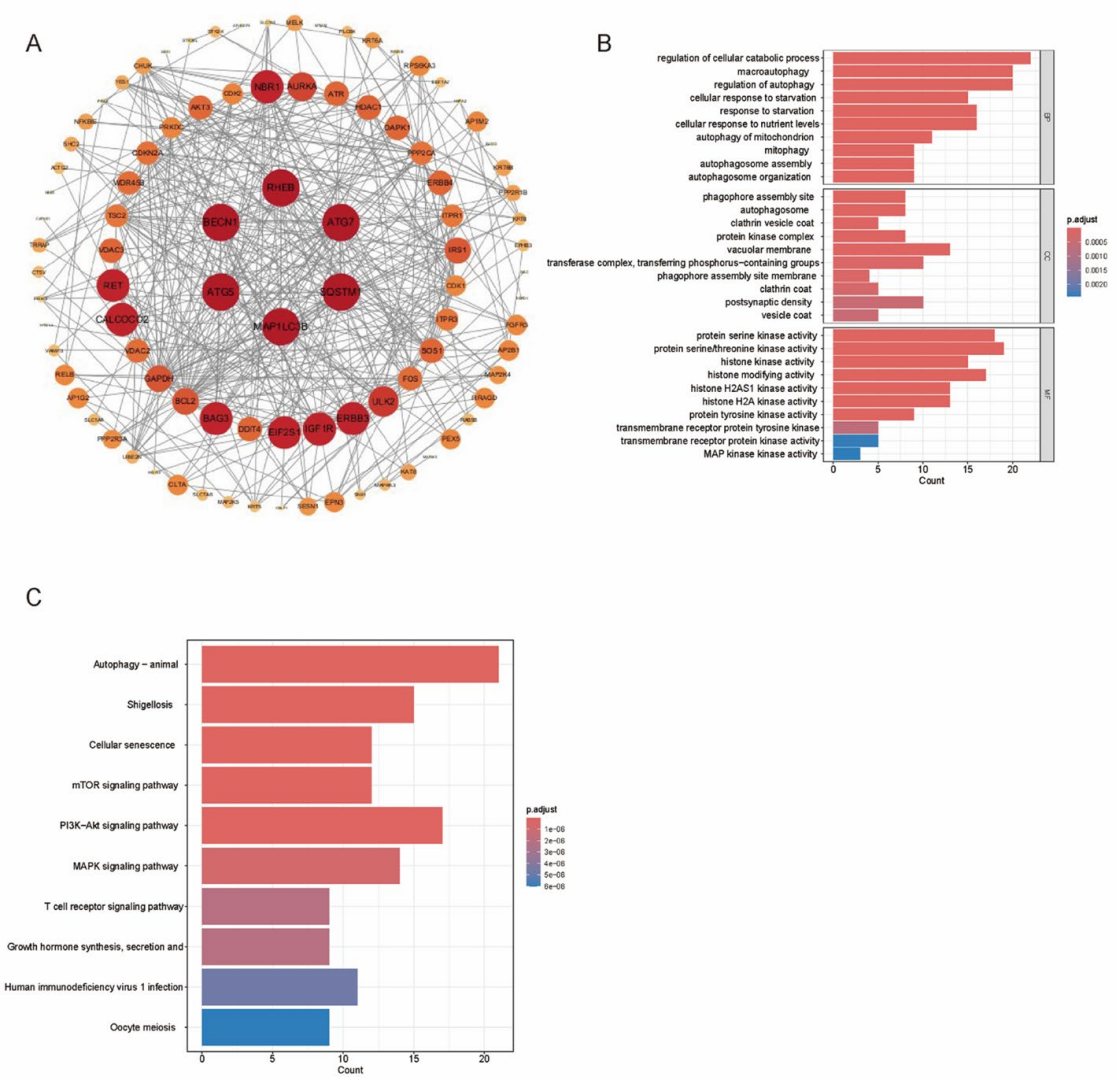
Features of TCGA-BRCA patients and IG expression. **A** PPI analysis of the 103 IG with high confidence; **B** GO enrichment of 103 IG. CC, cellular component; BP, biological process; MF, molecular function; **C** KEGG pathway enrichment of 103 IG

The PPI network of 103 IGs is displayed in Fig. 3A. As revealed by the GO analysis, 103 IGs were enriched primarily in the regulation of autophagy, regulation of cellular catabolic process, autophagy of mitochondrion, and cellular response to starvation (Fig. 3B). As revealed by the KEGG analysis, all IGs were enriched in autophagy - animal, cellular senescence, mTOR, PI3K-Akt, MAPK, and T cell receptor pathway (Fig. 3C).

### A C-ARG-based prognostic model

**Fig. 4:**
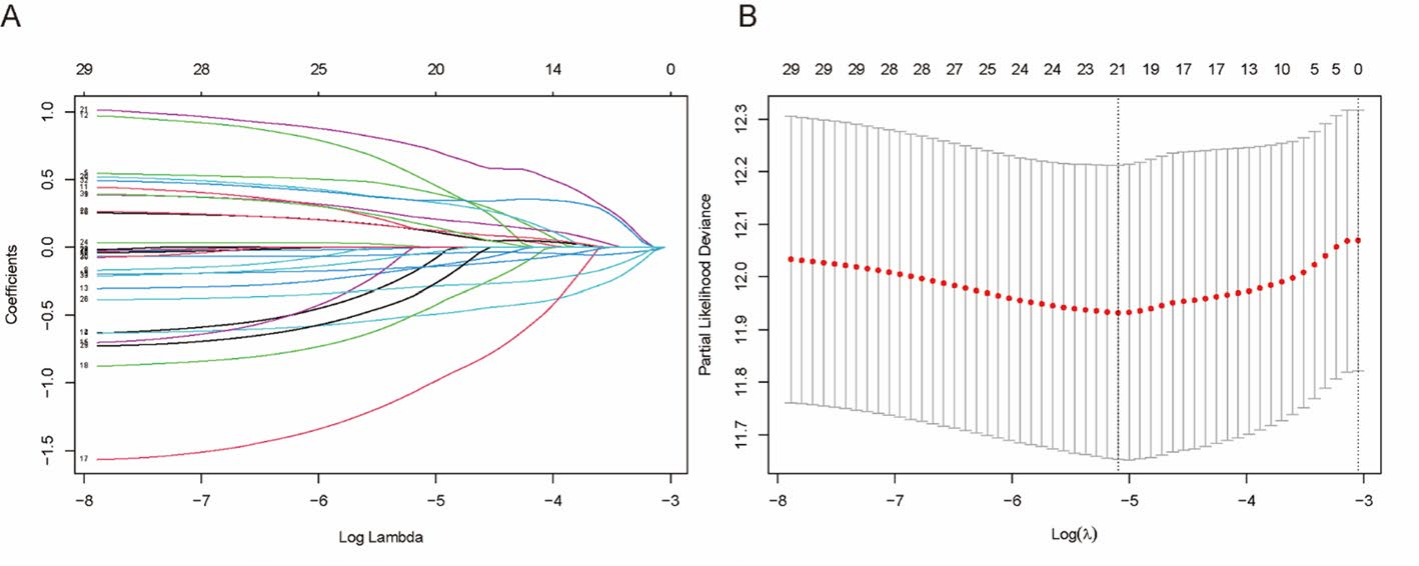
C-ARGs prognostic signature. **A**, **B** Lasso regression analysis

**Fig. 5:**
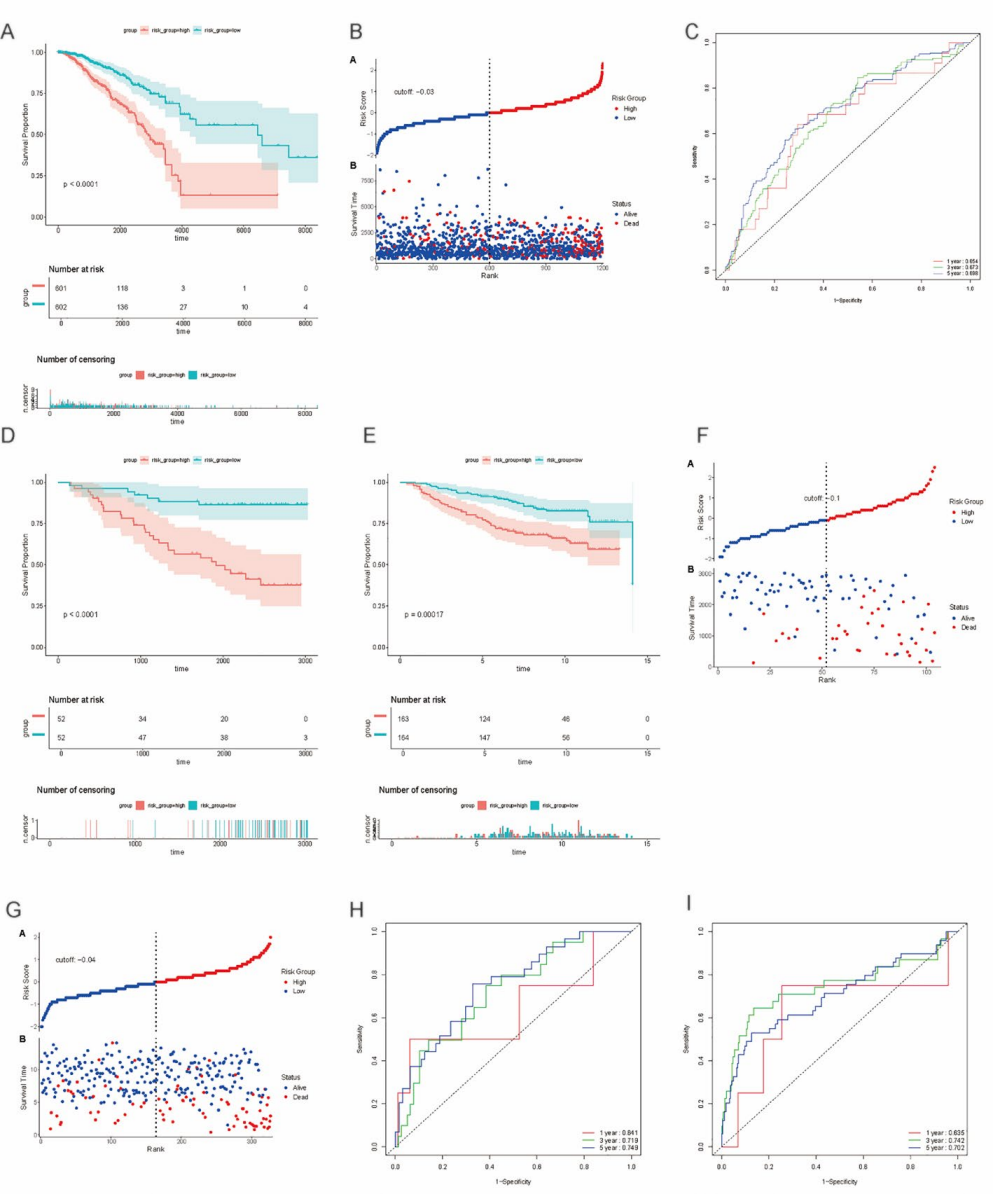
Performance of the 11 C-ARGs in the training and validation cohorts. **A** Kaplan‒Meier curves for OS in the high- and low-risk groups in the TCGA cohort; **B** Distribution of the risk scores and scatter plots for the survival status in the TCGA cohort; **C** ROC curves in the TCGA cohort; **D** Kaplan‒Meier curves for OS in the high- and low-risk groups in the GSE42568 cohort; **E** GSE20685 cohort; Distribution of the risk scores and scatter plots for the survival status in the **F** GSE42568 cohort; **G** GSE20685 cohort; ROC curves in the **H** GSE42568 cohort; **I** GSE20685 cohort

Univariate Cox analyses were conducted and identified 33 prognosis-related C-ARGs (*P* < .05). Then we further screened 21 genes by LASSO regression (Fig. 4A&B). Finally, 11 C-ARGs (*CLTA*, *HSPA2*, *SLC1A4*, *AKT3*, *DAPK1*, *ITPR1*, *MAP1LC3B*, *MAP2K5*, *RPS6KA3*, *ATG5*, and *DDIT4*) were utilized to create the prognostic model (Table 1). Based on the median of risk score, the TCGA-BRCA samples were assigned to high-/low-risk groups; longer OS was observed in the latter (*P* < .0001, Fig. 5A), and deaths increased and survival time decreased as the risk score rose in the two groups (Fig. 5B). Then, the model was assessed for the predictive accuracy using the ROC curve. The training cohort had AUCs of 0.654, 0.673, and 0.698, respectively, for 1-/3-/5-year OS (Fig. 5C), suggesting a moderate predictive power of the model.

**Table 1:** 11 C-ARGs and their coefficients

Gene	Coefficient
ATG5	0.6133193
SLC1A4	− 0.6874181
AKT3	0.3259830
CLTA	− 1.6605562
HSPA2	− 0.2025702
MAP2K5	0.5354045
MAP1LC3B	1.2689648
ITPR1	0.2934825
DDIT4	− 0.3774879
RPS6KA3	− 0.6872626
DAPK1	0.3208104

**Table 2:** Univariate and multivariable analyses for clinical characteristics

Dependent: surv (time, status)		All	HR (univariate)	HR (multivariate)
Race	Mean ± SD	2.7 ± 0.8	1.03(0.86–1.24, *p*=.715)	
Gender	Mean ± SD	0.0 ± 0.1	0.57(0.08–4.06, *p*=.573)	
Age	Mean ± SD	58.0 ± 13.1	1.03(1.02–1.04, *p*<.001)	1.04(1.02–1.05, *p*<.001)
Stage	Mean ± SD	2.1 ± 0.7	1.57(1.36–1.82, *p*<.001)	1.33(1.03–1.73, *p*=.031)
T	Mean ± SD	1.9 ± 0.7	1.30(1.11–1.52, *p*=.001)	0.96(0.79–1.17, *p*=.696)
N	Mean ± SD	0.8 ± 1.0	1.51(1.34–1.71, *p*<.001)	1.31(1.09–1.56, *p*=.004)
M	Mean ± SD	0.3 ± 0.7	1.11(0.88–1.39, *p*=.373)	
Riskscore	Mean ± SD	1.2 ± 0.9	1.50(1.37–1.63, *p*<.001)	1.49(1.36–1.62, *p*<.001)

Race, Gender, Age, Stage, T Tumor, N Lymph node, M Metastasis, HR Hazard ratio

Additionally, GSE42568 and GSE20685 were utilized for external validation. The high-risk group showed an unfavorable prognosis (*P* < .0001, Fig. 5D and E). Figure 5F and G displays the risk score and survival time. In the validation cohort, the AUCs for 1-/3-/5-year OS were 0.641, 0.719, and 0.749, respectively, in GSE42568 (Fig. 5H), and 0.635, 0.742, and 0.702, respectively, in GSE20685 (Fig. 5I).

### Construction of a nomogram

**Fig. 6:**
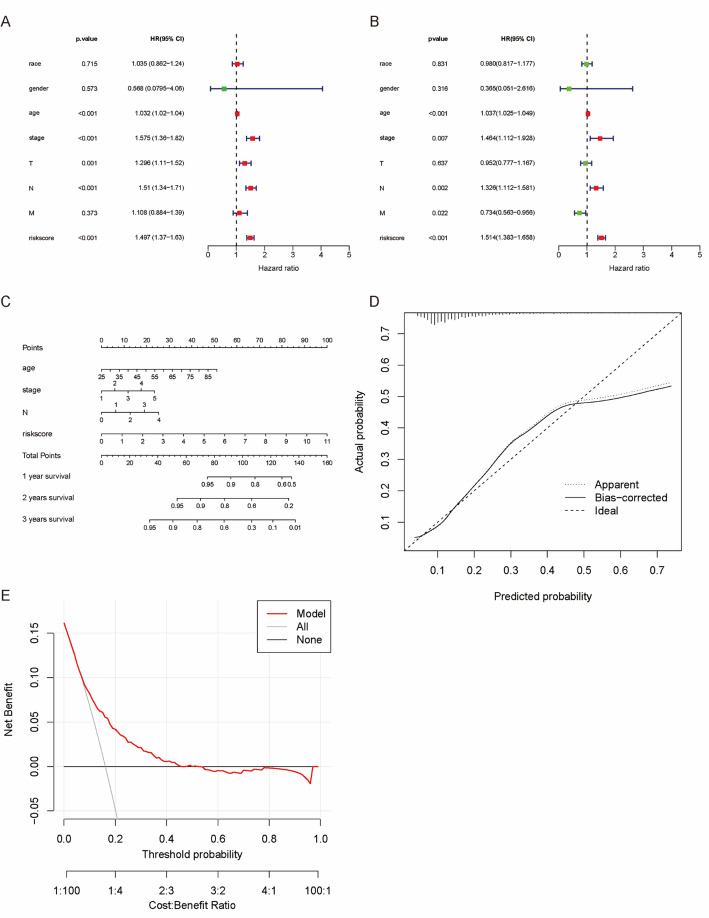
Independent prognostic predictors of the assessment model in the TCGA cohort **A** Forest plot for univariate analysis; **B** Forest plot for multivariate analysis; **C** Nomogram for 1-, 3-, and 5-year OS; **D** Calibration curves of the nomogram for 1-, 3-, and 5-year OS. **E** DCA

Univariate Cox analyses revealed significant differences in the age, risk score, stage, T, and N in the TCGA cohort (Fig. 6A), and these factors, except for T, still had great differences in multivariate analyses (Fig. 6B) (Table 2).

Based on age, stage, N, and risk score, we created a nomogram for 1-/3-/5-year OS (Fig. 6C), with a concordance index of 0.761. As demonstrated by both calibration curves and DCA, the nomogram was reliable in prognostic prediction (Fig. 6D&E).

### Immune infiltration and immune function analyses

**Fig. 7:**
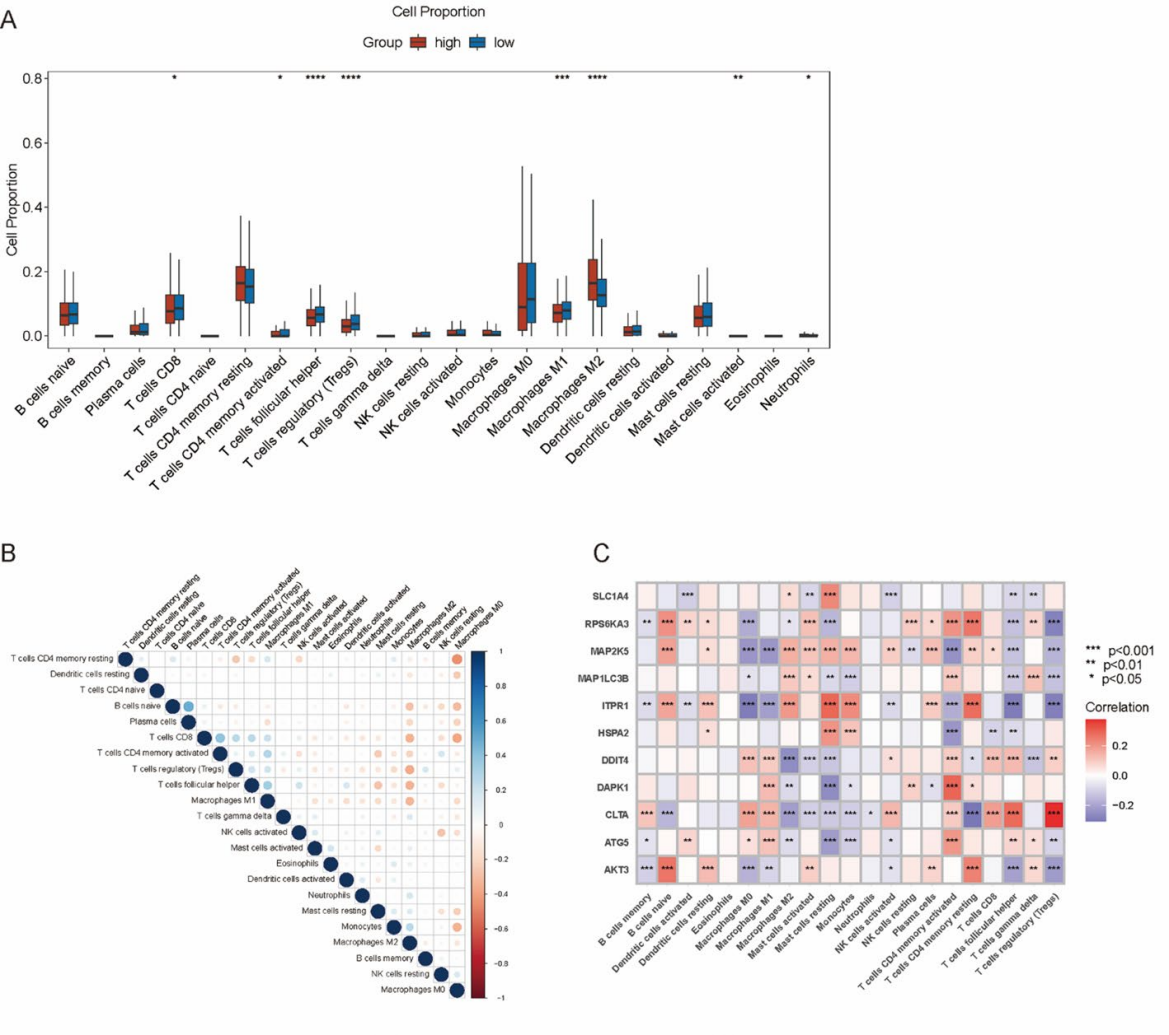
Tumor immune factors in the TCGA cohort. **A** Immune checkpoints; **B** Correlation heatmap of different immune cells and the risk scores. **C** Heatmap for the correlations between the expression of 11 C-ARGs and 22 infiltrating immune cells.* denotes *p* < .05; ** denotes *p* < .01;*** denotes *p* < .001

Furthermore, CIBERSORT was utilized to compare the status of immune cell infiltration in the TCGA-BRCA samples. The high-risk group had lower expressions of T cell CD4 memory activated, T cell CD8, T cell regulatory, T cell follicular helper, Macrophages M0, and Mast cells resting, but higher expressions of Macrophages M2 and Neutrophils (Fig. 7A). Moreover, a significant positive correlation was present in the infiltration of T cell CD8, T cell CD4 memory resting, and Macrophages M0, and T cell regulatory, Macrophages M2, and T cell follicular helper (Fig. 7B). In addition, we observed a close link between *CTLA* and infiltration of T cell follicular helper and T cell regulatory, between *ITPR1* and infiltration of T cell CD4 memory resting and Mast cells resting, and between *AKT3* and infiltration of B cells naïve and T cell CD4 memory resting (Fig. 7C).

Single-sample GSEA on the 11 C-ARGs revealed that *ATG5* was primarily enriched in the T cell receptor pathway, *HSPA2* was enriched in the chemokine pathway, *MAP1LC3* was enriched in Ras, cGMP-PKG, and Calcium pathways, *MAP2K5* was enriched in MAPK and Hedgehog signaling pathways, and *SLC1A4* was enriched in the Fanconi anemia pathway. All of these pathways were up-regulated (Fig. S1).

We assessed the relation of risk score with TME using ESTIMATE and found no statistically significant differences in the Tumor Purity and Immune/Stromal/ESTIMATE Scores (Fig. S2).

**Fig. 8:**
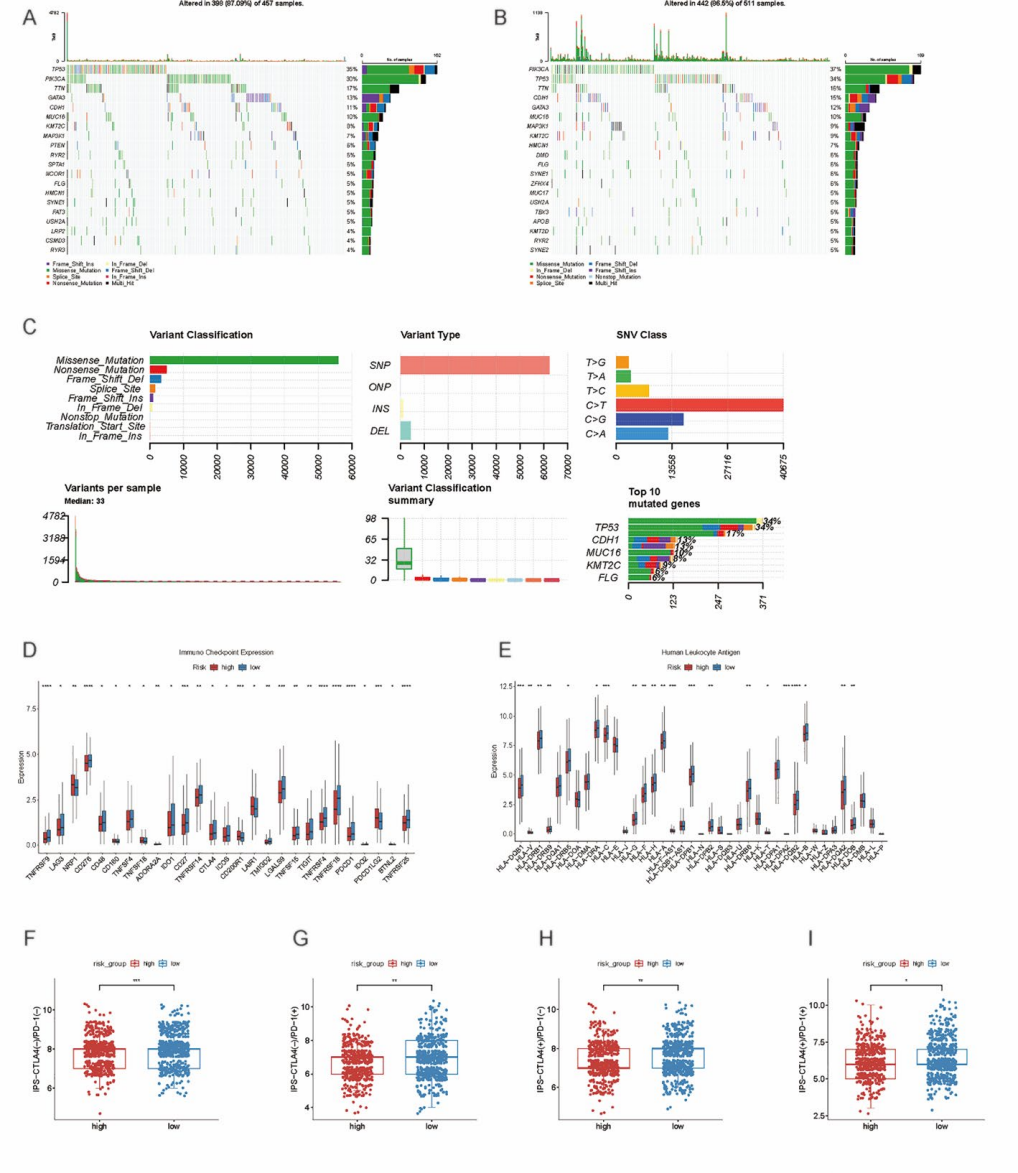
Immune signature and genomic features of C-ARG-based model. **A**, **B** Waterfall plot for the somatic mutation rate in high- and low-risk groups. Each column is an individual sample. The upper histogram: tumor mutation burden for each sample. The right histogram: percentage of each variant type. The number on the right reflects the mutation frequency for each gene; **C** According to different classification categories, missense mutation, SNP, and C > T mutation occupied a higher proportion, Mutation burden in each sample. The summary of the occurrence of each variant classification. Top 10 mutated genes in BRCA. SNP, single-nucleotide polymorphism; SNV, single nucleotide variant; **D** Immune checkpoints; **E** Human Leukocyte Antigen; **F**–**I** Relative distribution of IPS based on CTLA-4 or PD-1 in the high- and low-risk groups in TCGA cohort.* denotes *p* < .05; ** denotes *p* < .01;*** denotes *p* < .001

The tumor mutational burden (TMB) score can be used for monitoring the efficacy of tumor immunotherapy. Therefore, to clarify whether the risk score correlates with the BRCA mutation profiles, we compared the somatic mutation profile (Fig. 8A-C). *PIK3CA* and *TP53* were identified to have the highest frequency of mutation.

In addition, ICs and HLA were compared. The results revealed that the low-risk group had high expressions of *PDCD1*, *CTLA-4*, *CD48*, and *TNFRS4* receptors, while the expressions of *CD160*, *LAIR1*, and *CD200R1* rose in the high-risk group (*P* < .05) (Fig. 8D). An obvious difference was detected in HLA in the two groups (Fig. 8E). These findings suggest the model’s predictive value in the immune response of BRCA to immunotherapy. Besides, the IPS analysis manifested that the low-risk group was superior in the immunotherapy effect regardless of the immunophenotype of *PD1* and *CTLA-4*. This indicates to some extent that low-risk patients have higher sensitivity to immunotherapy so that their survival time can be further extended (Fig. 8F-I). Furthermore, the level of regulatory T cells (Tregs) was upregulated in the high-risk group.

### Model performance in predicting drug sensitivity

**Fig. 9:**
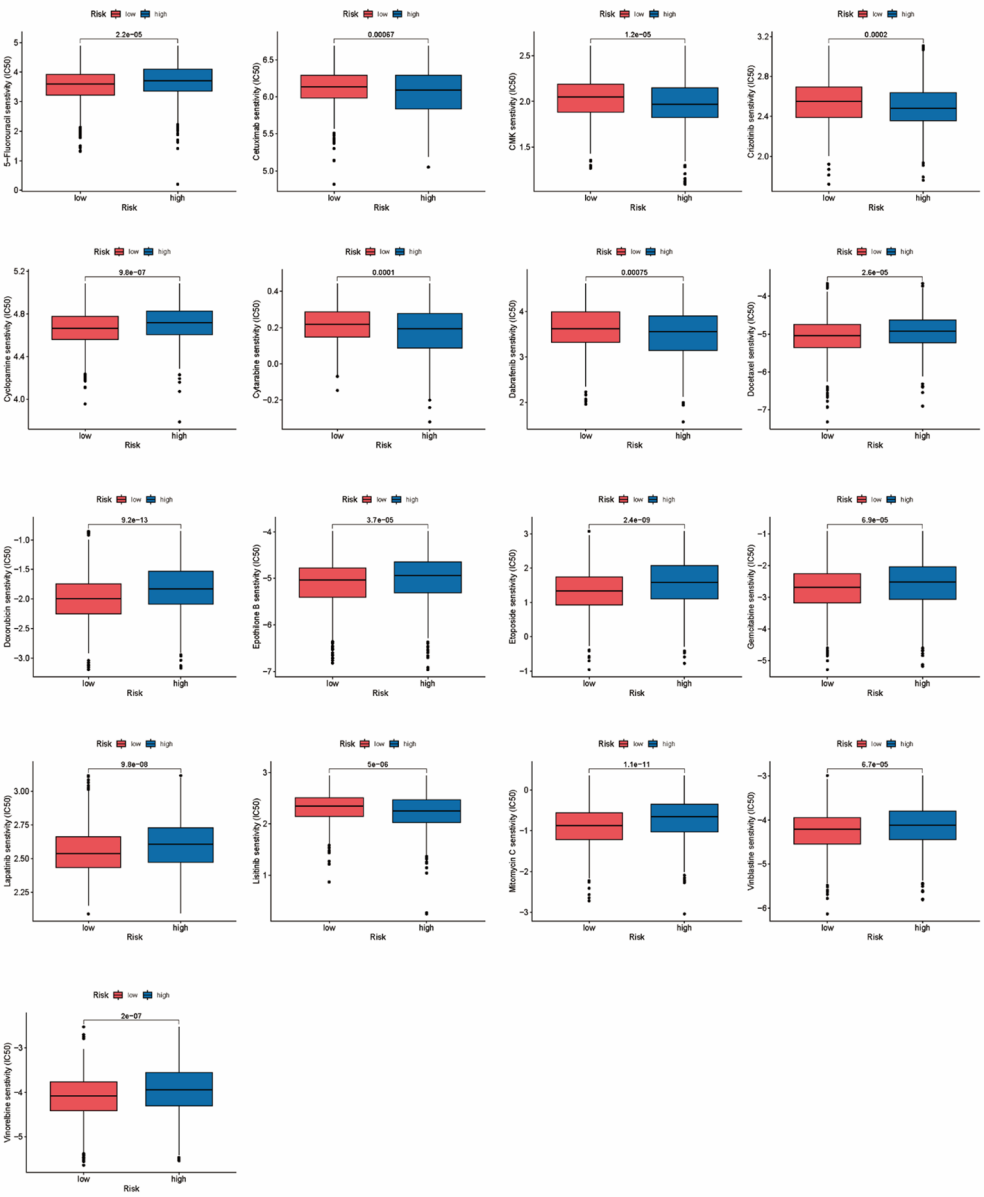
Comparison of IC50 in the two groups

To further clarify the model’s value in the BRCA treatment, we assessed the chemotherapeutic effect on BRCA using the pRRophetic package, and drug sensitivity was compared between the two groups by Wilcoxon signed-rank tests. The high-risk group exhibited higher sensitivity to fluorouracil, vinorelbine, vinblastine, gemcitabine, etoposide, doxorubicin, docetaxel, cetuximab, mitomycin C, lapatinib, epothilone B, and cyclopamine than the low-risk group (*P* < .001). In contrast, the low-risk group showed higher sensitivity to linsitinib, dabrafenib, crizotinib, cytarabine, and CMK (Fig. 9). To sum up, the high-risk patients have higher drug sensitivity.

### IHC staining

**Fig. 10:**
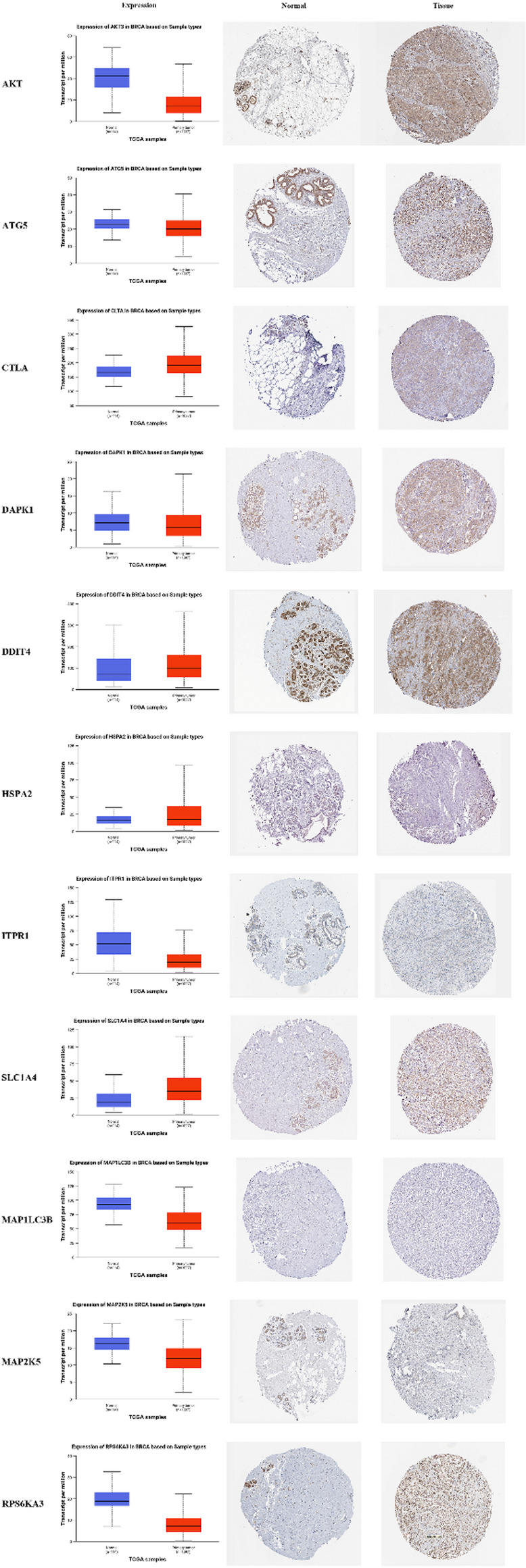
Comparison of gene expression and IHC images in normal and tumor tissues

To assess the protein expression of C-ARGs involved in the model, the IHC results from the HPA were compared with the data on gene expression in the TCGA. We found that *CLTA*, *HSPA2*, and *SLC1A4* were highly expressed in BRCA tissues but lowly expressed in normal breast tissues (*P* < .001, Fig. 10). *AKT3*, *DAPK1*, *ITPR1*, *MAP1LC3B*, *MAP2K5*, and *RPS6KA3* had high expressions in normal breast tissues and low expressions in BRCA tissues (*P* < .05). No statistical difference was detected in the *ATG5* (*P* = .2144) and *DDIT4* protein expressions (*P* = .056145) between the normal breast tissues and BRCA tissues.

## Discussion

BRCA is a common malignancy endangering female health globally [1]. With the advances in treatment, NAC has become a standard treatment for early high-risk patients, which can lower the stage and reduce the tumor volume to conserve the breast. Moreover, BRCA patients achieving pCR following NAC may experience longer DFS and OS, but some still respond poorly to NAC [2] and rarely benefit from NAC, which not only affects efficacy but may also lead to waste of medical resources and unnecessary toxic side effects. Therefore, accurately identifying patients sensitive to NAC has become one of the key issues in ameliorating the BRCA prognosis. With the rapid advance in personalized medicine, predictive biomarkers have become increasingly important, which can enable clinicians to accurately identify patients most likely to benefit from NAC, thereby developing individualized treatment protocols. In this way, appropriate patients may undergo targeted therapy, while chemotherapy-insensitive patients may be relieved from the burden of overtreatment or ineffective treatment, potentially contributing to more precise and efficient management of BRCA. Over the past decade, the importance and function of autophagy across cancers have dramatically changed, and its crucial role in the onset and progression of cancers (e.g., colon, pancreatic, and cervical cancers) has been documented [9, 10]. Furthermore, autophagy affects tumor cells’ tolerance to chemotherapeutic agents through multiple mechanisms (e.g., intracellular signaling pathways and tumor microenvironment regulation), thereby serving as a key molecular mechanism that impacts chemotherapy efficacy and chemoresistance [11, 12].Currently, most prognostic models based on ARGs for BRCA focus on predicting breast cancer recurrence and metastasis [13–15], however, studies exploring the role of ARGs in the chemosensitivity and prognosis of BRCA remain scarce.

Therefore, we constructed a C-ARG-based prognostic model that forecasts prognosis, drug sensitivity, and immune infiltration conditions, thereby facilitating more accurate therapeutic strategies for BRCA.

In this study, the prognostic model covered 11 C-ARGs (*ATG5*, *SLC1A4*, *AKT3*, *CLTA*, *HSPA2*, *MAP2K5*, *MAP1LC3B*, *ITPR1*, *DDIT4*, *RPS6KA3*, and *DAPK1*). The pivotal roles of these C-ARGs in the initiation, progression, metastasis, and chemoresistance of BRCA have been highlighted in numerous studies [16–30]. For example, a study involving 274 patients with breast infiltrating ductal carcinoma revealed that the cytoplasmic *SQSTM1* and *MAP1LC3B* proteins have high expressions in cancer tissues, the high co-expression of the two is linked to improved DFS, and autophagy inhibition affects the cancer cells’ sensitivity to chemotherapeutic agents [16]. In addition, different *AKT* isoforms vary in their function in BRCA. For example, *AKT1* contributes to tumor cell proliferation and survival and suppresses metastasis; *AKT2* mainly facilitates migration, invasion, and metastasis of tumor cells; *AKT3*, highly expressed in TNBC and *HER2* + BRCA, is highly related to BRCA recurrence [17]. Yidan Liang et al. (2021) [18] argued that cadmium exposure will restrain *ACSS2/ATG5*-mediated autophagy and facilitate BRCA cell proliferation, migration, and invasion, thus inducing BRCA development. By comparing the plasma cfDNA between 90 BRCA patients and 30 healthy controls, Esmat Ghalkhani (2021) held that plasma *DAPK1* gene promoter methylation levels are far higher in metastatic BRCA than in non-metastatic BRCA, which can serve as a potential diagnostic biomarker for BRCA [19]. *MAP2K5* plays a vital role in multiple critical processes of BRCA initiation, progression, and recurrence, including metastasis, cancer stem cell maintenance, and acquisition of drug resistance [20–25]. In TNBC, inhibiting *MAP2K* may contribute to enhanced anticancer effects of cytotoxic chemotherapeutics (e.g., docetaxel, vinorelbine, cisplatin) [26]. *MAP2K5* mRNA expression correlates with reduced recurrence-free survival in BRCA patients undergoing chemotherapy [27]. In addition, *DDIT4*, *ITPR1*, and *SLC1A4* have been verified to correlate with BRCA progression, efficacy, and immune microenvironment, and they are expected to be prognostic biomarkers and therapeutic targets [28–30]. To sum up, we can reasonably speculate that these genes may serve as potential biomarkers for chemotherapy responsiveness and prognosis in BRCA. Therefore, a C-ARG-based prognostic model was established for predicting the immune infiltration, prognosis, and drug sensitivity, achieving more precise treatment for BRCA of NAC.

Detailed analyses were made on the immune infiltration, prognosis, and drug sensitivity in both groups. The OS was longer in the low-risk group. The robustness of the model was validated using ROC curves. Following clinical parameter adjustment, the age, stage, risk score, and N were identified to be independent indicators for survival status. Taken together, these results imply that the model potentially exhibited good predictive power for BRCA prognosis.

Immune function analyses were conducted in both groups from the perspectives of immune infiltration, HLA, ICs, and TMB. HLA is important for connecting innate and adaptive immunity, which can recognize, process, and present protein antigens (e.g., pathogens) to relevant T cells and NK cells, initiating immune responses. Highly expressed HLA helps the immune system recognize tumor cells and enhances the immune cells’ killing effect on tumor cells [31]. Therefore, a stratified analysis was performed on HLA in TCGA. Most of the HLA antigens were up-regulated in the low-risk group, possibly suggesting that both the immune recognition function and survival outcomes were more favorable in this group.

As an emerging strategy, IC blockade can restore the anti-tumor immune response by inhibiting negative regulators for effector T cells, clearing tumor cells. *CTLA-4*, *PD-1*, *CD48*, and *TNFRS4* receptors, the most common biomarkers for IC inhibitors, interact with the ligands *CD274* (*PD-L1*), *B7-1* (*CD80*), *CD-2*, and *OX40L* to be involved in immune activation, immunoregulation, and maintenance of immune homeostasis [32–35]. In contrast, *LAG3*, *CD160*, and *LAIR1* play opposite roles [36–38]. This study revealed high levels of *CTLA-4*, *CD48*, *PD-1*, and *TNFRS4* receptors in the low-risk group, while *LAG3*, *CD160*, and *LAIR1* had high expressions in the high-risk group. Furthermore, the low-risk group was superior in the immunotherapy effect regardless of the immunophenotype of *PD1* and *CTLA-4*. This may suggest that low-risk patients had higher sensitivity to immunotherapy, potentially yielding a longer survival time. Contradictorily, the levels of Tregs were lower in the high-risk group than in the low-risk group. Elevation of Tregs, as key immunosuppressive cells, typically restrains antitumor immune responses, theoretically correlating with poorer immunotherapy outcomes [39]. However, the contrary results were obtained in this study. Combined with current research progress, it is hypothesized that the main reason for this contradiction lies in the significant immunodepletion of the immune system in the high-risk group. Specifically, effector immune cells in the high-risk group were chronically exposed to persistent tumor antigen stimulation, thus gradually losing proliferative capacity and cytotoxic function and becoming insensitive to immune response signals [40]. Therefore, despite lower levels of Tregs observed in the high-risk group, it may be difficult for immunotherapy to activate effective antitumor immune responses potentially due to a state of “functional failure” in effector cells, ultimately producing less favorable clinical outcomes. However, its mechanism requires further experimental validation. To sum up, C-ARGs may offer new predictors for immunotherapy for BRCA.

However, some deficiencies are worth noting. First, this study was conducted based on the existing datasets, so the model remains to be further validated in a large cohort. Second, due to few available data in TCGA, we failed to fully analyze the BRCA subtypes, which may also account for the non-positive results in some immunoassays. Third, this study lacked in vitro and in vivo experimental validation, with no functional experiments or animal models, limiting mechanism interpretation and translational potential. To address these deficiencies, we should prospectively collect detailed data on BRCA subtypes and long-term follow-up records in large clinical studies to validate and optimize the C-ARG-based model, design in vitro experiments to explore target C-ARG functions, and perform in vivo validation via nude mouse xenograft models to confirm the effects of C-ARGs on tumor progression in the future. In this way, the clinical applicability of the C-ARG-based model can be strengthened.

## Conclusion

In conclusion, the novel C-ARG-based prognostic model for BRCA demonstrates promising predictive performance in both the TCGA training cohort and GEO validation cohort. Low-risk BRCA patients may tend to have more favorable immune infiltration profiles and IC expression patterns. Based on these observations, the model can potentially provide valuable reference information for guiding NAC and immunotherapy for BRCA patients.

## Supplementary Information

Below is the link to the electronic supplementary material.

Supplementary Material 1. Fig. S1 GSEA of 11 genes, Fig. S2 TME

## Data Availability

The datasets generated during and/or analysed during the current study are available in the GEO (https://www.ncbi.nlm.nih.gov/geo/) and TCGA (https://www.cancer gov/geo/). The codes used in this article are available from the original author by request.
