# Supplementary figures and images for: Autophagy-related gene-based prognostic model for breast cancer
Source: Discov Oncol. 2026 Mar 7;17:565. doi: 10.1007/s12672-026-04771-1 (PMC13083393; doi:10.1007/s12672-026-04771-1)

**Supplementary Materials**

**
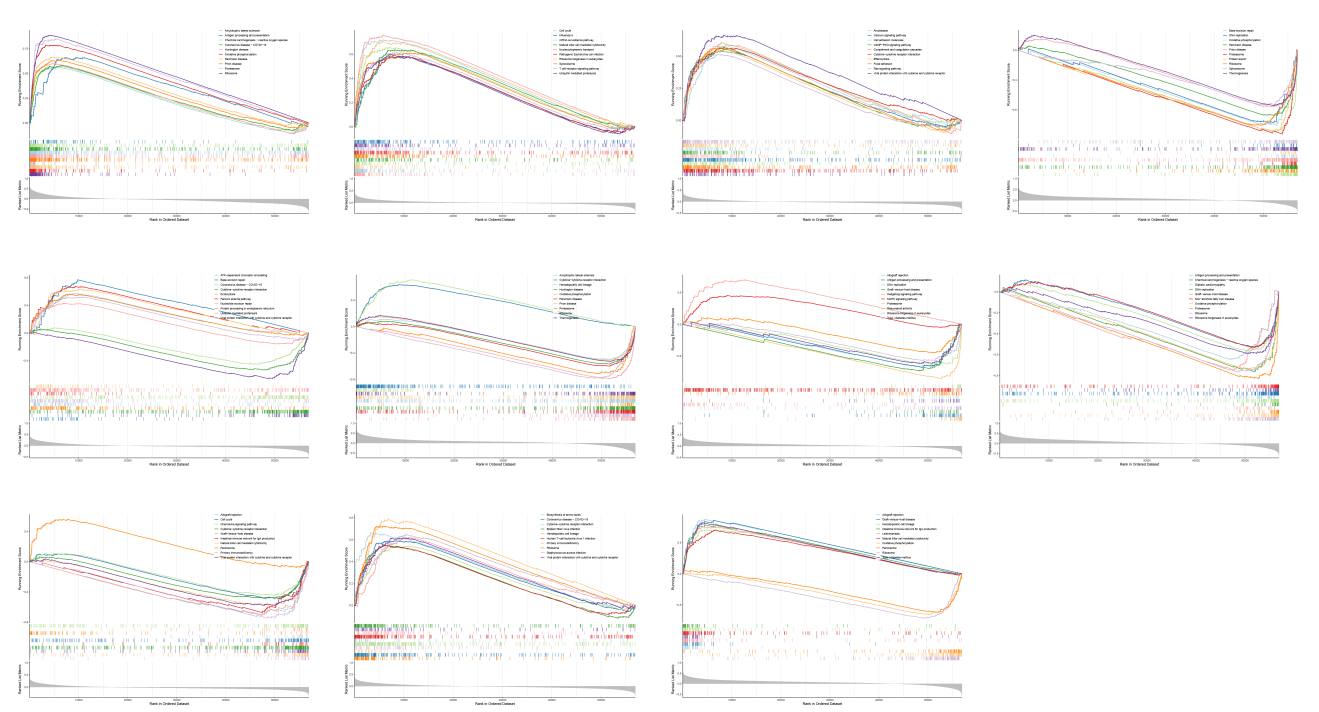
**

**Fig. S1** GSEA of 11 genes


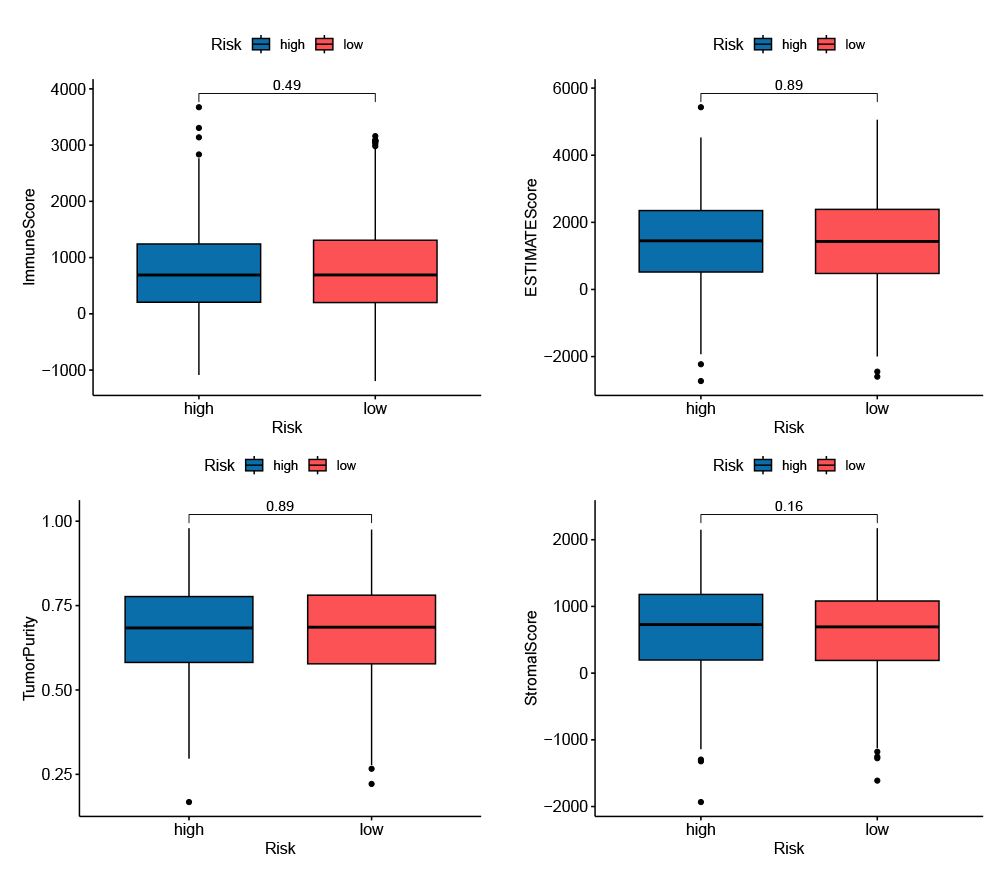


**Fig. S2** TME

Supplement: Supplementary file 1 — Supplementary Material 1. Fig. S1 GSEA of 11 genes, Fig. S2 TME [file 12672_2026_4771_MOESM1_ESM.docx]
